# Identification of immune characteristic landscapes related to autophagy in ischemic stroke

**DOI:** 10.3389/fcell.2022.1026578

**Published:** 2022-11-29

**Authors:** Shuang Li, Yu Zhang, Shanshan Shi, Da Guo, Ting Chang

**Affiliations:** ^1^ Department of Neurology, Tangdu Hospital, The Fourth Military Medical University, Xi’an, Shaanxi, China; ^2^ Department of Neurosurgery, Tangdu Hospital, The Fourth Military Medical University, Xi’an, Shaanxi, China; ^3^ Department of Neurology, The First Affiliated Hospital, Harbin Medical University, Harbin, Heilongjiang, China; ^4^ Honghui Hospital, Xi’an Jiaotong University, Xi’an, Shaanxi, China

**Keywords:** autophagy, immune characteristic, ischemic stroke, correlation analysis, weighted gene co-expression network analysis (WGCNA)

## Abstract

Ischemic stroke (IS) is a common and grievous nervous system disease. Both autophagy activation and immune response after cerebral ischemia play important roles in the development of IS. Many studies have revealed a close interplay between autophagy and immunity. However, little is known about how autophagy influences the immune characteristics of IS. Hence, the study aims to systematically explore the role of autophagy and its impact on immune characteristics in IS. We first compared the expression differences of autophagy genes in a training set and identified 20 dysregulated autophagy genes between healthy and IS samples. An autophagy-related classifier composed of seven genes was further established and could well distinguish healthy and IS samples. Then, the association between autophagy and immune characteristics, including infiltrating immunocytes, activity of immune reactions, and HLA gene expression, was investigated. The results showed that autophagy closely correlated with immune characteristics, such as NAMPT and ARNT significantly related to infiltrating immunocytes; PPP1R15A and CASP3 significantly related to activity of immune reactions; and NAMPT and ATG16L2 significantly related to HLA genes. Next, two distinct autophagy expression patterns were identified by unsupervised clustering analysis, and diverse immune characteristics were discovered between them. A total of 5481 autophagy phenotype-related genes were obtained between two expression patterns, and their biological functions revealed that these genes were involved in immune-related biological pathways. Finally, five dysregulated autophagy genes (*FOS*, *MAP1LC3B*, *ERO1L*, *ARNT*, and *PPP1R15A*) were proved between IS and healthy samples using another two validation sets. Our results illustrated that autophagy had a dramatic effect on the immunity of IS and provided a novel sight into understanding the pathogenesis of IS.

## Introduction

Ischemic stroke (IS), one of the most common and devastating diseases, is a leading cause of death and disability worldwide (Collaborators GN Global, 2019). It usually occurs due to the disruption of the blood supply to some brain regions, thus resulting in neuron death or permanent neurological deficit (Hossmann, 2006). Inflammation and immunity have been demonstrated to be key elements of the pathophysiology of stroke (Iadecola and Anrather, 2011). The inflammation process, an integral part of the ischemic cascade, starts at the intravascular compartment immediately after arterial occlusion, while circulating innate immune cells are quickly activated at the onset of arterial occlusion, ultimately causing invasion of the ischemic brain by blood-borne immune cells and activation of brain-resident cells (Iadecola and Anrather, 2011; Iadecola et al., 2020). Microglial cells, the resident macrophages of the brain, are highly activated after brain injury. Studies suggest that microglial cells may limit post-stroke inflammation by phagocytizing dead cells and neutrophils, generating neurotrophic factor IGF-1 and inhibiting astrocyte activation, and the depletion of microglial cells worsens stroke outcomes (Lalancette-Hébert et al., 2007; Jin et al., 2017; Otxoa-de-Amezaga et al., 2019). Neutrophils are the earliest immune cells recruited into the ischemic brain, which contribute to ischemic insult by releasing proteases and forming neutrophil extracellular traps (Iadecola et al., 2020). In addition to innate immune cells, adaptive immune cells (T and B cells) also exert critical roles in the mechanism of IS (Qin et al., 2020). However, post-stroke immunity is a double-edged sword, which can be either beneficial or detrimental, and is worth exploring deeply.

Recent studies illustrate that autophagy plays a crucial role in the pathogenesis of IS, and regulation of the autophagy activity may affect the outcome of IS (Shi et al., 2021). Autophagy is a dynamic process of self-degradation of intracellular components mediated by multiple lysosomal enzymes, through which unnecessary or dysfunctional components, including certain long-lived proteins, insoluble proteins, and impaired organelles, are eliminated to maintain cell homeostasis (Klionsky, 2008). Based on the method of cargo delivery to the lysosomes, autophagy is classified into three different types, namely, microautophagy, macroautophagy, and chaperone-mediated autophagy (Wang et al., 2018). Generally, moderate autophagy exerts a neuroprotective effect in IS by regulating neural survival and death with variable mechanisms (Wang et al., 2018). For example, in a vessel occlusion mouse model, the autophagy activator rapamycin could reduce the infarction volume and improve motor deficits (Hwang et al., 2017). Nevertheless, IS often triggers maladaptive autophagy. Excessive or persistent activation of autophagy is detrimental to neurons by way of activation of cell death mechanisms (Ajoolabady et al., 2021). Upregulation of IL-2 in the ischemic brain tissues could promote autophagy gene expression in neuronal cells after hypoxia/ischemia and contribute to neuronal injury (Clarkson et al., 2014). Therefore, the double role autophagy plays in the pathogenesis of IS remains to be explored.

There is a complex crosstalk relationship between the autophagy pathway/proteins and immunity. In recent years, growing evidence has demonstrated that autophagy is involved in many immune processes, such as autoantigen presentation, cytokine production, and survival of lymphocytes, suggesting an apparent and vital role in the innate and adaptive immune responses (Yin et al., 2018). For instance, the autophagy pathway can activate type I IFN production in plasmacytoid dendritic cells by delivering viral nucleic acids to endosomal Toll-like receptors (Lee et al., 2007). Accumulating studies have shown that both autophagy and immunity exert an important role in the process of cerebral ischemic injury, and there is a close interplay between autophagy and immunity. Although autophagy- or immunity-related IS has been investigated, most of them only concentrate on one molecule or pathway, and comprehensively systematic research on autophagy in IS and how autophagy affects immune characteristics are still not uncovered.

Taken together, in our study, we systematically portrayed the roles of autophagy and the related immune characteristics in IS. We first found that the autophagy gene classifier can well distinguish healthy and IS samples. Immune characteristics, including infiltrating immunocytes, activity of immune reactions, and HLA gene expression, were then observed to be a significant correlation with autophagy. Next, we clustered IS samples using autophagy genes and identified two different autophagy-mediated regulation patterns. Furthermore, the two patterns were found to possess diverse clinical and immune characteristics. Functional enrichment and WGCNA analysis were performed to investigate the biological functions and the key module of autophagy-related expression patterns, respectively. These results indicated that autophagy regulation patterns have significant implications on the immune characteristics of IS.

## Materials and methods

### Data acquisition

Three gene expression datasets used in the present study, including GSE22255 (20 IS vs. 20 healthy samples), GSE58294 (69 IS vs. 23 healthy samples), and GSE16561 (39 IS vs. 24 healthy samples), were downloaded from the Gene Expression Omnibus (GEO) database (https://www.ncbi.nlm.nih.gov/geo/). Details of the selected datasets are presented in Supplementary Table S1. The GSE22255 (Krug et al., 2012) profile was used to identify dysregulated autophagy genes and analyze the related immune characteristics, while the GSE58294 (Stamova et al., 2014) and GSE16561 (Barr et al., 2010) datasets were applied for the validation of results. Data processing was performed using R (version 4.1.3). During data processing, probes were annotated as gene symbols using a platform annotation file. Gene probes were excluded, which had multiple matching gene symbols or were without matching gene symbols. As for duplicate gene symbols, the median value was selected as the expression value. The 222 autophagy genes investigated in this study were acquired from the Human Autophagy Database (HADb, http://www.autophagy.lu/).

### Alteration analysis of autophagy genes between IS and healthy samples

The differentially expressed autophagy genes between IS and healthy samples were analyzed using the “Empirical Bayes method” in the R package “limma”. Genes with *p* < 0.05 and |foldchange (FC)| > 1 were considered significant dysregulated autophagy genes. Pearson correlation coefficient (PCC) was used to evaluate the expression relationship of the dysregulated autophagy genes in all samples and IS samples. The protein–protein interaction (PPI) network of dysregulated autophagy genes was constructed through STRING v11.0 (Szklarczyk et al., 2019) and then visualized with Cytoscape software. Univariate logistic regression was applied to identify IS-related autophagy genes with the cutoff criteria of *p* < 0.05. Then, the LASSO (least absolute shrinkage and selection operator) regression was performed for feature selection and dimension reduction. Furthermore, the autophagy classifier of IS was developed using multivariate logistical regression. Finally, the distinguishing performance of the signature was assessed through the receiver operating characteristic (ROC) curve analysis.

### Correlation analysis between autophagy genes and immune characteristics

Single-sample gene-set enrichment analysis (ssGSEA) defines an enrichment score to represent the degree of absolute enrichment of a gene set in each sample within a given data set (Barbie et al., 2009). In this study, we evaluated the infiltrating immunocytes and the activity of immune reactions through ssGSEA analysis. According to a previous study (Shen et al., 2019), we first obtained the gene sets used to assess the enrichment degree of the infiltrating immunocytes. The gene sets related to immune reactions were then downloaded from the ImmPort database (http://www.immport.org) (Bhattacharya et al., 2014). The enrichment scores of the infiltrating immunocytes and immune reaction activity and the expression of the HLA genes were compared by the Wilcoxon test between IS patients and healthy controls. The correlation of autophagy genes with immunocyte fractions, immune reaction activity, and HLA gene expression was calculated using PCC analysis.

### Analysis of autophagy gene expression patterns

Unsupervised clustering analysis was performed to identify different autophagy gene expression patterns based on the expression of 222 autophagy genes. The cluster numbers and robustness were estimated using a consensus clustering algorithm (Chai et al., 2019; Zhang et al., 2020). The robustness of classification was guaranteed using the R package “ConsensusClusterPlus” with the aforementioned steps for 1000 iterations. PCA was further conducted to verify the expression status of 222 autophagy genes in distinct expression patterns. The differences in infiltrating immunocytes, immune reactions, and HLA gene expression between distinct expression patterns were compared by a *t-*test. A chi-squared test was conducted to compare the clinical characteristics of two expression patterns.

### Identification of autophagy phenotype-related genes and gene modules

To obtain autophagy phenotype-related genes, differentially expressed genes between two expression patterns were analyzed by the empirical Bayesian approach of the R package “limma”. The criterion of significant differentially expressed genes was set as the *p*-value < 0.05. Weighted gene co-expression network analysis (WGCNA) by the “WGCNA” package in R (Langfelder and Horvath, 2008) was used to identify the autophagy phenotype-related gene modules through the following major steps. A cluster of all samples was performed to check if any obvious outliers existed. A co-expression network was then constructed based on the matrix of pairwise Pearson correlation coefficients using the WGCNA method. The dynamic tree cut algorithm with min was applied to cluster genes into different functional modules with different colors. Module membership (MM) and gene significance (GS) were calculated to correlate modules with the phenotype. Finally, after extracting the important module gene information, it was used for further analysis.

### Analysis of biological functions for distinct autophagy expression patterns

To portray the biological functions of two expression patterns, the KEGG pathway enrichment analysis was conducted. The gene sets for “c2.cp.kegg.v7.5.1.symbols” were acquired from the Molecular Signatures Database (MSigDB) (Liberzon et al., 2011). The GSVA (gene-set variation analysis) algorithm was performed to transform the expression matrix of the gene sets to the pathway activation score matrix. The Wilcoxon test was applied to compare the pathway activation score matrix of two expression patterns, and the cut-off criterion was set as *p*-value < 0.05. Meanwhile, GO-BP enrichment analysis of autophagy phenotype-related genes was employed using the R package “clusterProfiler” (Yu et al., 2012). GO-BP with a *p*-value < 0.05 was considered to be a significantly enriched functional annotation.

### Validation of dysregulated autophagy genes using GEO datasets

As mentioned previously, we obtained two additional datasets (GSE58294 and GSE16561) from GEO to validate the dysregulated autophagy genes. The “Empirical Bayes method” in the R package “limma” was performed again to identify the differentially expressed autophagy genes in GSE58294 and GSE16561, respectively, with *p* < 0.05 and |FC| > 1 as the threshold. Ultimately, the intersection genes among GSE22255, GSE58294, and GSE16561 were regarded as high-confidence differentially expressed autophagy genes.

## Results

### The landscape of autophagy genes between IS and healthy samples

**FIGURE 1:**
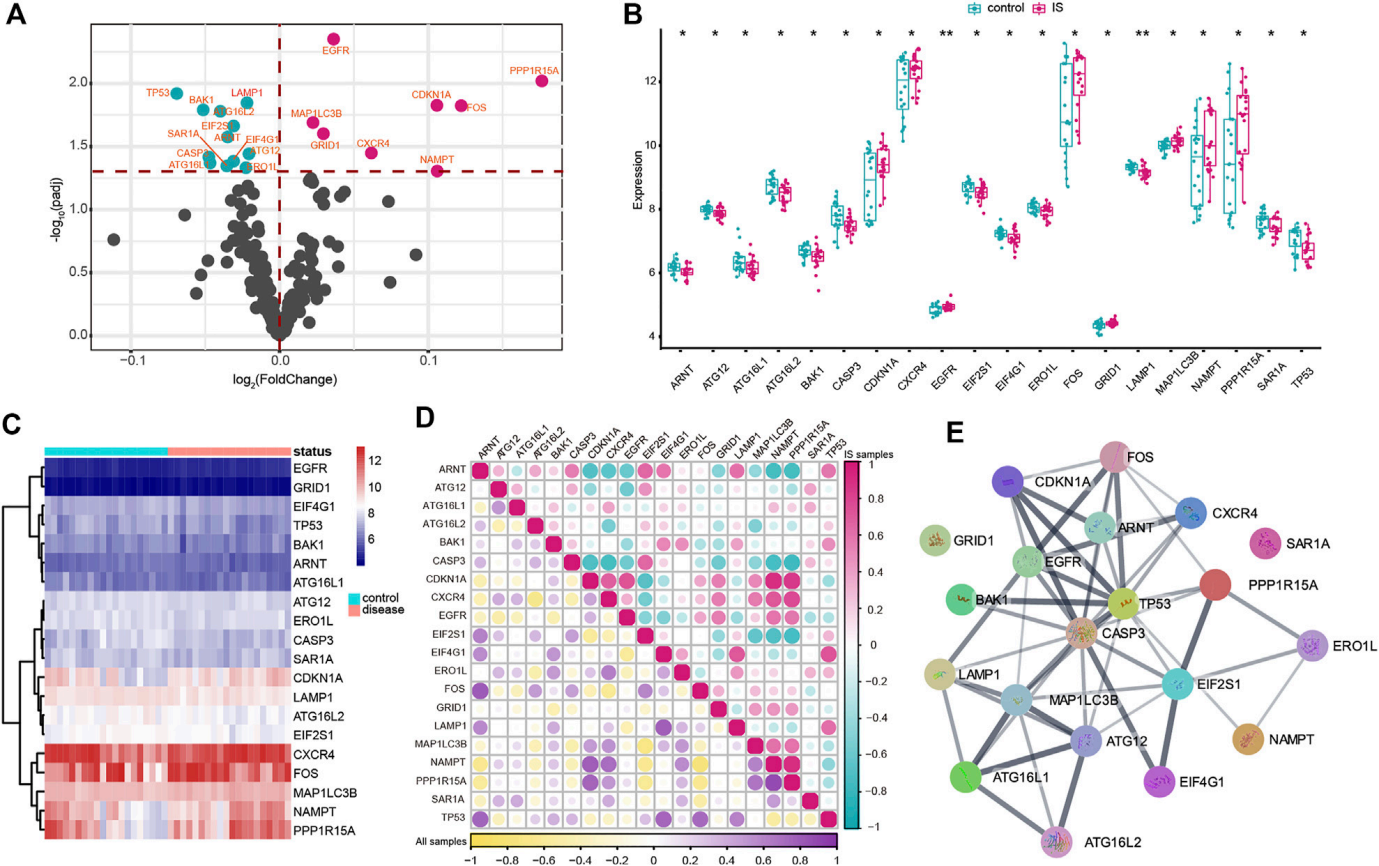
Expression landscape of autophagy genes in IS. **(A)** Volcano plots of differentially expressed autophagy genes. Purple circles represented upregulated genes, and green circles represented downregulated genes. **(B,C)** Box plot and heatmap plot showed the expression status of 20 dysregulated autophagy genes between IS and healthy samples. **(D)** Correlation analysis among 20 significantly dysregulated autophagy genes in all samples and IS samples. **(E)** Interactions among multiple proteins encoded by dysregulated autophagy genes.

In the study, 222 autophagy genes were acquired from HADb to explore autophagy alteration status in IS. The differential analysis identified 20 dysregulated autophagy genes associated with IS, namely, *ARNT*, *ATG12*, *ATG16L1*, *ATG16L2*, *BAK1*, *CASP3*, *CDKN1A*, *CXCR4*, *EGFR*, *EIF2S1*, *EIF4G1*, *ERO1L*, *FOS*, *GRID1*, *LAMP1*, *MAP1LC3B*, *NAMPT*, *PPP1R15A*, *SAR1A*, and *TP53* (Figure 1A). Meanwhile, the transcriptome expression status of the significantly dysregulated autophagy genes between IS and healthy samples is represented in Figures 1B,C. To reveal the relationships among the dysregulated autophagy genes, we performed correlation analysis and found that *PPP1R15A* and *NAMPT* were the most correlated autophagy genes (Figure 1D). In addition, the protein–protein network was constructed to portray the dysregulated autophagy gene interactions (Figure 1E), and we noticed that *CASP3*, *TP53*, and *MAP1LC3B* might exert more important roles in the network. Enrichment analysis revealed that the biological features of 20 dysregulated autophagy genes were related to cellular response to the external stimulus, cellular response to the extracellular stimulus, and cellular response to the abiotic stimulus (Supplementary Table S2).

### Construction of the autophagy-related classifier to distinguish IS and healthy samples

**FIGURE 2:**
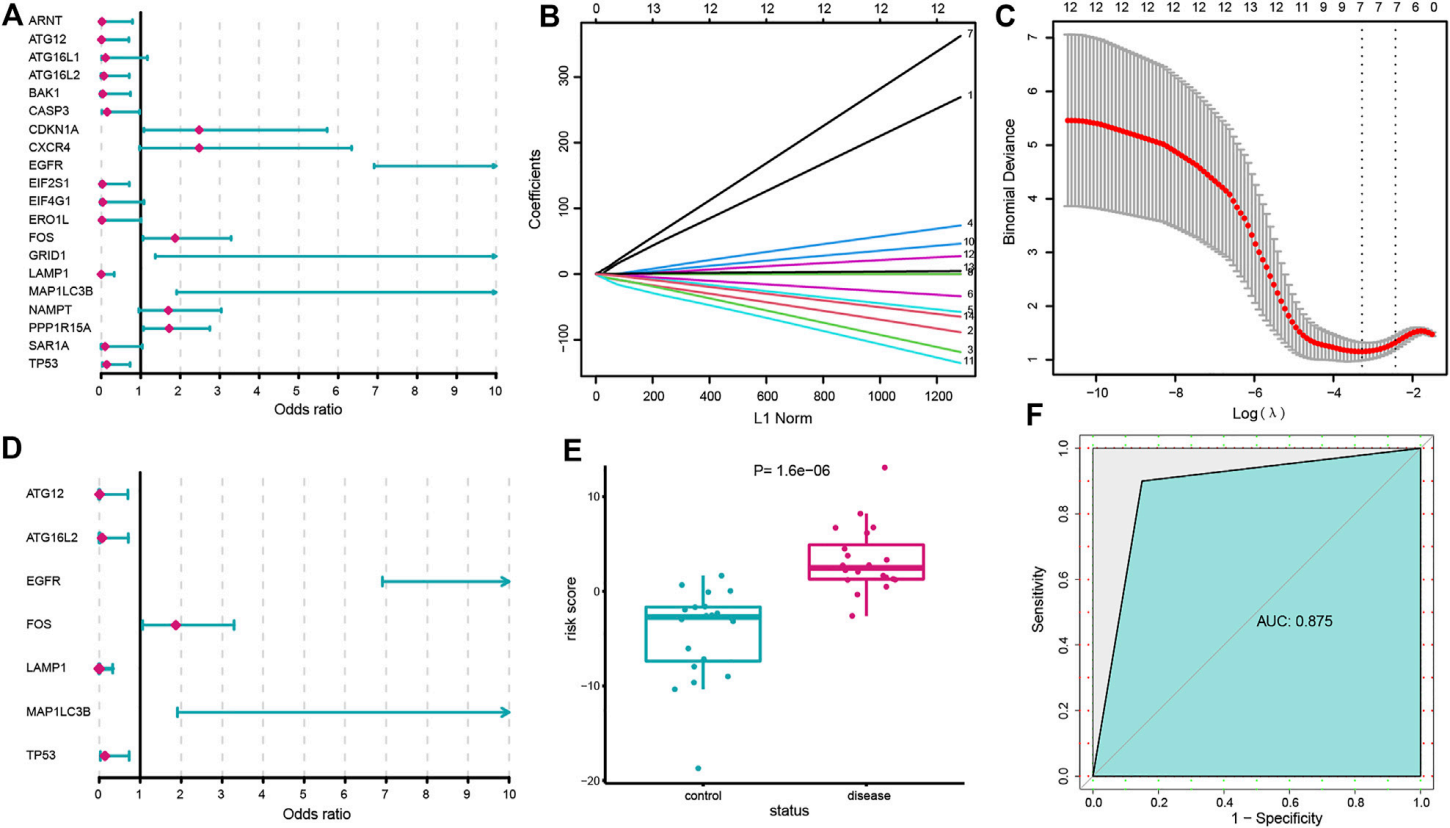
Construction of the autophagy-related classifier. **(A)** Relationships between 20 dysregulated autophagy genes and IS using univariate logistic regression analysis. **(B)** Least absolute shrinkage and selection operator (LASSO) coefficient profiles of dysregulated autophagy genes (*p* < 0.05). **(C)** 10-fold cross-validation for tuning parameter selection in the LASSO regression. The partial likelihood deviance is plotted against log (λ), where λ is the tuning parameter. Partial likelihood deviance values are shown, with error bars representing SE. The dotted vertical lines are drawn at the optimal values by minimum criteria and 1-SE criteria. **(D)** Multivariate logistic regression developed a classifier consisting of seven autophagy genes. **(E)** Risk distribution between IS and healthy subjects, where IS has a much higher risk score than healthy samples. **(F)** ROC curve evaluated the discrimination ability of the classifier model.

A series of bioinformatic algorithms were conducted on the 20 dysregulated autophagy genes to identify the IS-associated crucial features. We first employed univariate logistic regression analysis to find out the relationships between the dysregulated autophagy genes and IS (Figure 2A) and found that 14 autophagy genes (*EGFR*, *LAMP1*, *TP53*, *MAP1LC3B*, *PPP1R15A*, *ATG16L2*, *EIF2S1*, *FOS*, *BAK1*, *CDKN1A*, *ATG12*, *ARNT*, *GRID1*, and *CASP3*) were significantly correlated with IS (*p* < 0.05). Then, LASSO regression was used for feature selection and dimension reduction on the 14 dysregulated autophagy genes and 7 essential autophagy genes (*ATG12*, *ATG16L2*, *EGFR*, *FOS*, *LAMP1*, *MAP1LC3B*, and *TP53*) for IS were screened (Figures 2B,C). The seven vital autophagy genes were further passed onto a multivariate logistic regression analysis for classifier construction (Figure 2D). Finally, we obtained risk scores for each of the samples, and the results demonstrated that the autophagy-related classifier consisted of seven crucial features that can well distinguish healthy and IS samples (Figure 2E), where IS had a much higher autophagy risk score than healthy samples (*p* < 0.0001). The ROC curve was plotted, and it indicated that the autophagy-related classifier model has excellent discrimination ability (Figure 2F). Meanwhile, we validated the performance of the autophagy-related classifier using the GSE58294 dataset and obtained a similar result, which suggested that the classifier model possessed an outstanding robustness (Supplementary Figure S1).

### The correlation between autophagy and immune characteristics of IS

**FIGURE 3:**
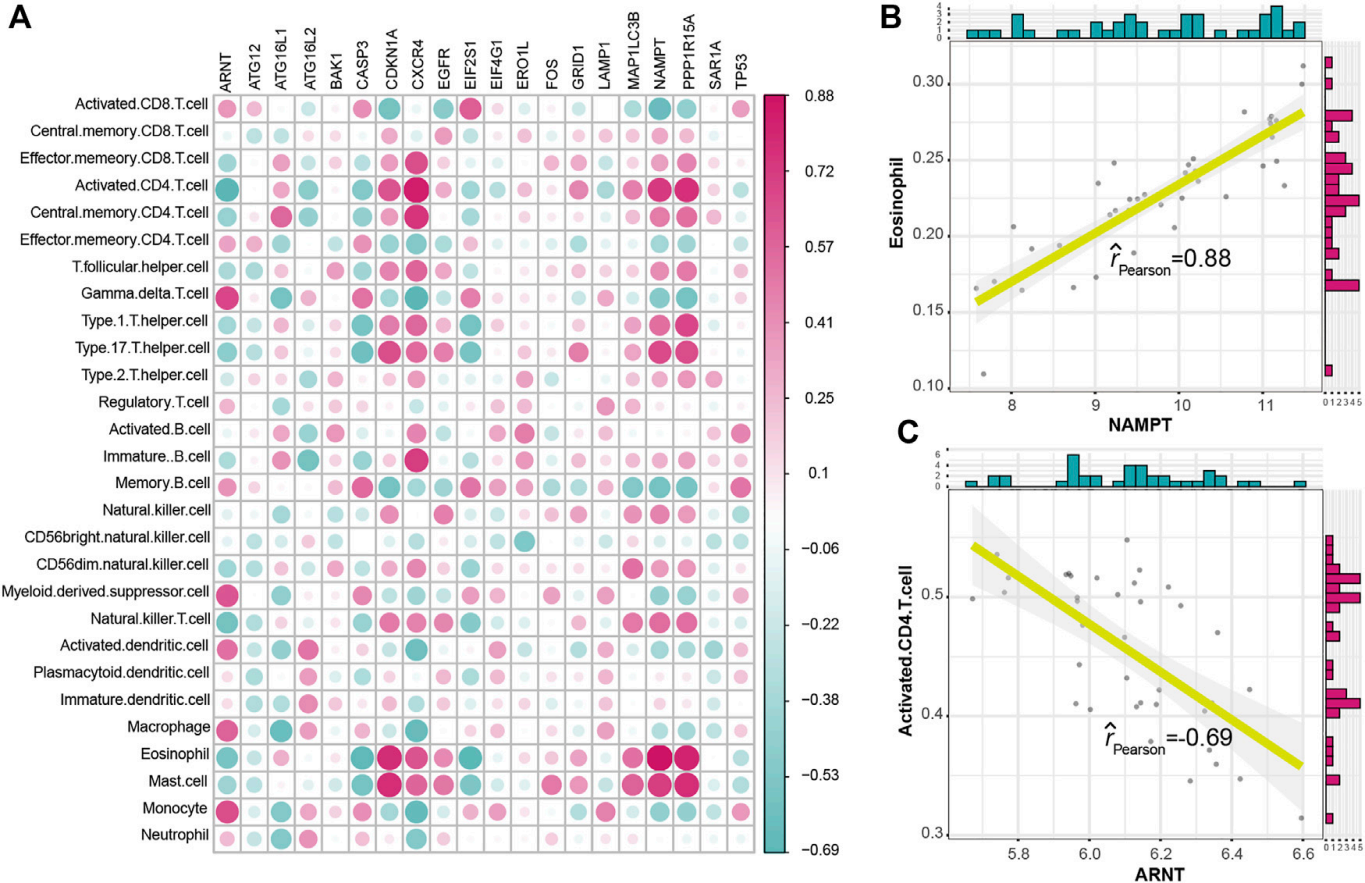
Correlation between immunocyte cells and dysregulated autophagy genes. **(A)** Dot plot showed the correlations between each immunocyte cell type and each dysregulated autophagy gene. **(B)** Most positively correlated immunocyte–autophagy gene pair. **(C)** Most negatively correlated immunocyte–autophagy gene pair.

**FIGURE 4:**
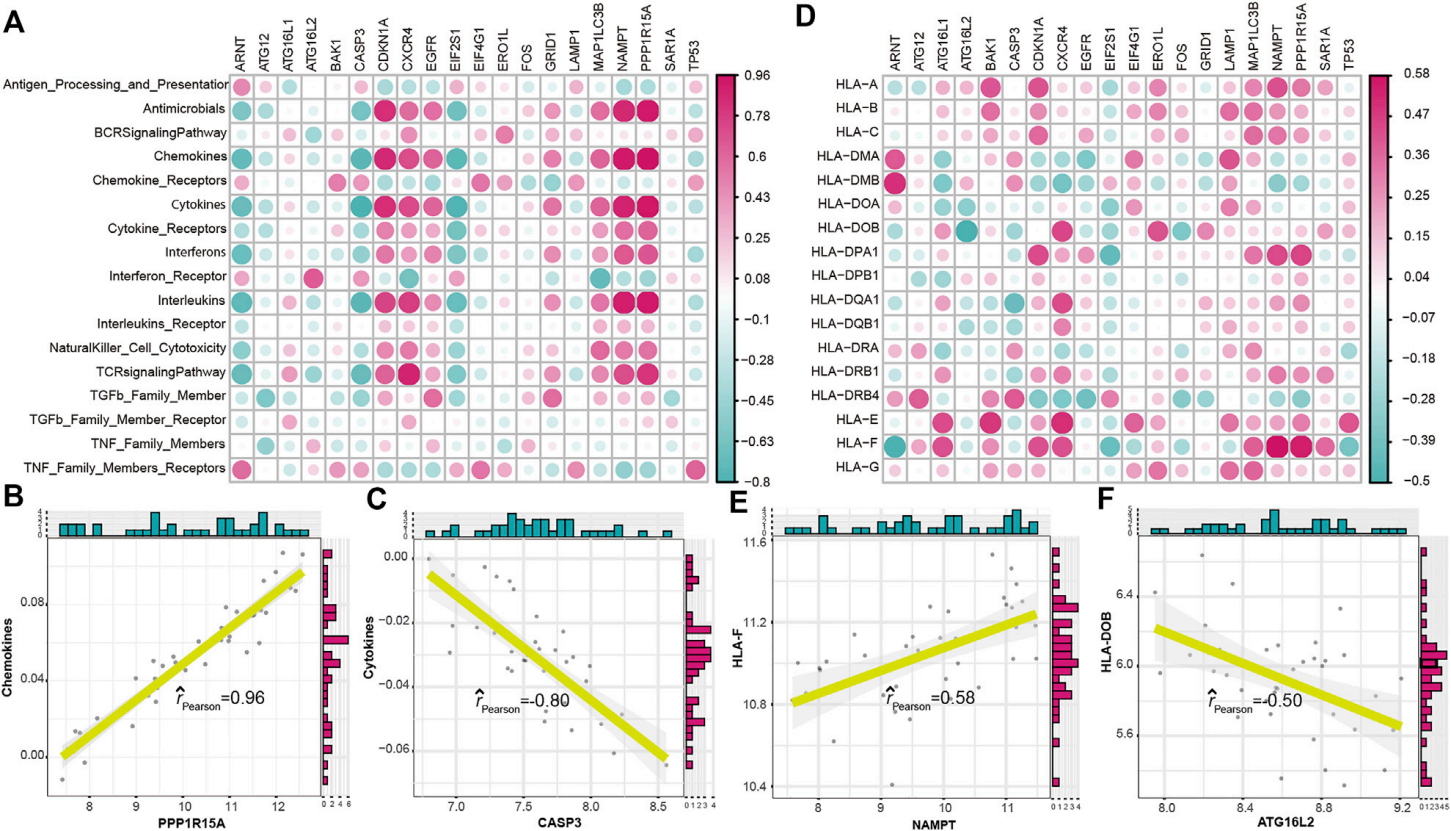
Correlation between immune reactions or HLA genes and dysregulated autophagy genes. **(A)** Dot plot showed the correlations between each immune reaction and each dysregulated autophagy gene. **(B)** Most positively correlated immune reaction–autophagy gene pair. **(C)** Most negatively correlated immune reaction–autophagy gene pair. **(D)** Dot plot showed the correlations between each HLA gene and each dysregulated autophagy gene. **(E)** Most positively correlated autophagy–HLA gene pair. **(F)** Most negatively correlated autophagy–HLA gene pair.

To explore the relationship between autophagy and immune characteristics in IS, a correlation analysis was performed for autophagy genes with infiltrating immunocytes, immune reactions, and HLA gene expression referred to in a previous study (Zhang et al., 2021). ssGSEA was employed to calculate the enrichment abundance of each immunocyte between IS and healthy samples (Supplementary Figure S2). Some of the immunocyte fractions changed in the IS samples, such as memory B cells, natural killer cells, and mast cells. We further investigated the correlation of the infiltrating immunocytes with autophagy genes and found that dysregulated autophagy genes were closely related to multiple immunocytes (Figure 3A). The most positively correlated immunocyte-autophagy gene pair was eosinophil–NAMPT, and a higher expression of NAMPT and a higher score of eosinophil were found in IS (Figure 3B), while the most negatively correlated pair was activated CD4 T cell-ARNT, and a lower expression of ARNT and a higher level of activated CD4 T-cell population could be found in IS (Figure 3C). Likewise, the activity of immune reactions and the expression levels of HLA genes were analyzed in IS. Several significant changes in the immune reaction activity were observed between IS and healthy samples (Supplementary Figure S3), such as chemokines, cytokines, and TGF- β family members increased, while TNF family member receptors decreased in IS. The correlations between immune reactions and autophagy genes were then calculated and are presented in Figure 4A. PPP1R15A–chemokines was the most positively correlated pair, and CASP3-cytokines was the most negatively correlated pair (Figures 4B,C). The results illustrated that PPP1R15A and CASP3 might play an important role in the immune reactions of chemokines and cytokines, respectively. For the expression status of HLA, although no significant difference was found between the IS and healthy samples (Supplementary Figure S4), the correlation with dysregulated autophagy genes remained (Figure 4D). The most positively correlated autophagy-HLA pair was NAMPT–HLAF (Figure 4E), while the most negatively correlated autophagy–HLA pair was ATG16L2–HLADOB (Figure 4F).

### Different expression patterns of autophagy genes in IS

**FIGURE 5:**
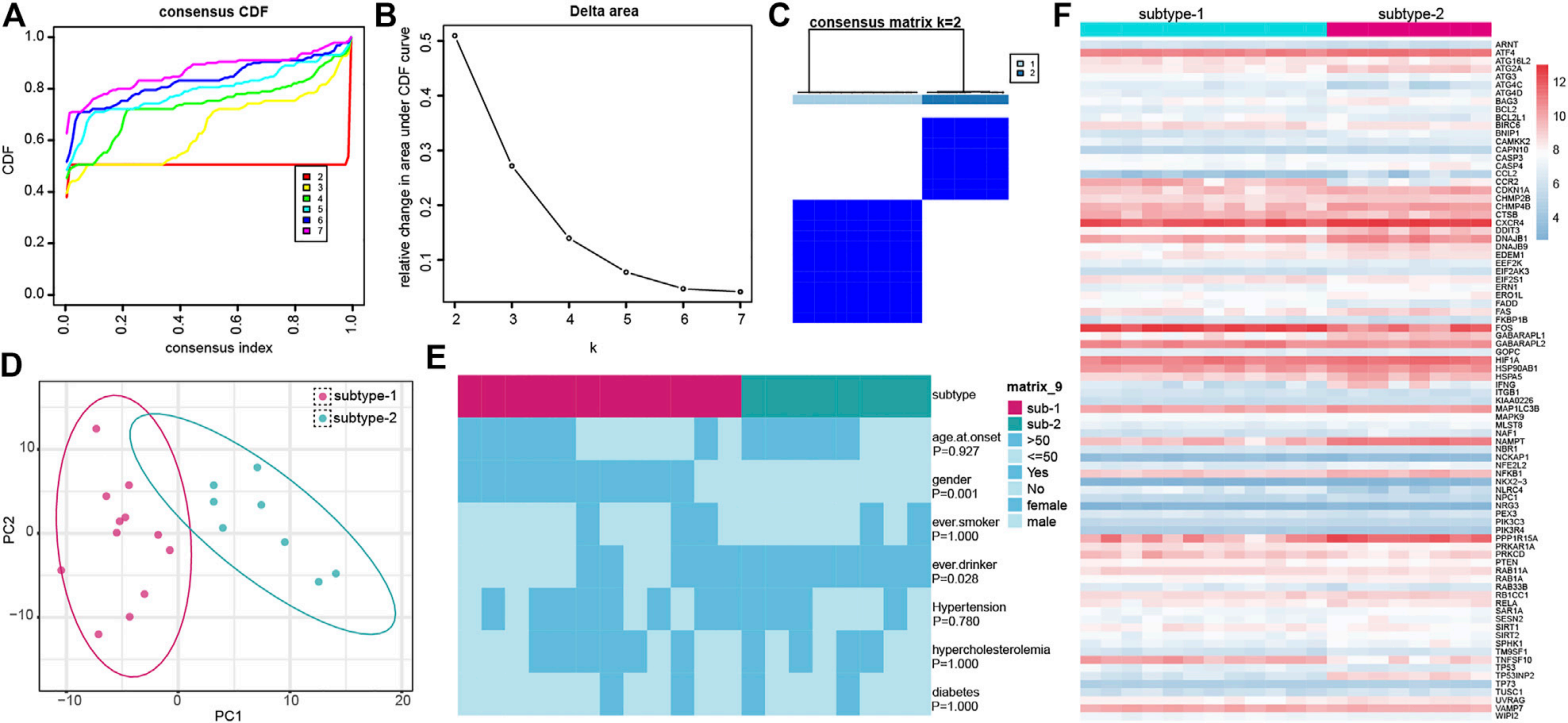
Identification of different autophagy gene expression patterns by unsupervised consensus clustering. **(A)** Consensus clustering cumulative distribution function (CDF) for k = 2–7. **(B)** Relative change in area under the CDF curve for k = 2–7. **(C)** Heatmap of the matrix of co-occurrence proportions for IS samples. **(D)** PCA analysis for the transcriptome profiles of the two distinct autophagy subtypes. **(E)** Comparison of clinical characteristics between the two subtypes. **(F)** Expression status of subtype-specific autophagy genes in the two subtypes.

To further identify the regulation status of autophagy in IS, unsupervised consensus clustering analysis was applied for the IS samples based on the expression of 222 autophagy genes (Figures 5A–C). As a result, we obtained two distinct subtypes of IS, including 12 samples in subtype-1 and 8 samples in subtype-2. The detailed information on the patients and subtypes of IS is shown in Supplementary Table S3. A PCA of the two subtypes revealed that there was a dramatic difference in transcriptome expression between the two patterns (Figure 5D). Then, the clinical characteristics between the two subtypes were compared (Figure 5E). Gender (*p* = 0.001) and overdrinking (*p* = 0.028) were found to be different between the two subtypes. Next, we compared the expression of autophagy genes in the two subtypes and found 84 subtype-specific autophagy genes significantly changed between the two subtypes, unsupervised clustering of which demonstrated distinct expression patterns in the two subtypes (Figure 5F).

### Immune characteristics in the two distinct autophagy expression patterns

**FIGURE 6:**
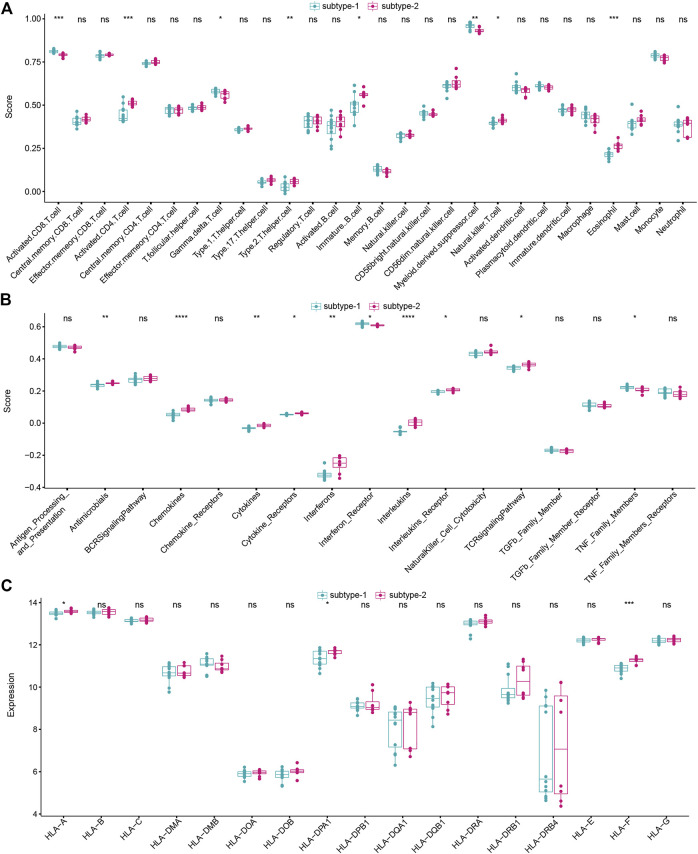
Different immune characteristics of two distinct autophagy subtypes. **(A)** Abundance differences of each immune immunocyte in two autophagy expression subtypes. **(B)** Activity differences of each immune reaction in two autophagy expression subtypes. **(C)** Expression differences of each HLA gene in two autophagy expression subtypes.

To figure out the immune characteristics of the two subtypes, infiltrating immunocytes, immune reactions, and the expression of HLA were compared. As expected, different immune characteristics were observed between the two distinct autophagy expression patterns. For instance, higher levels of activated CD8 T cells, γδ -T cells, and myeloid derived suppressor cells were enriched in subtype-1, while higher levels of activated CD4 T cells, type-2 T helper cells, immature B cells, natural killer T cells, and eosinophils were enriched in subtype-2 (Figure 6A). As for immune reactions, subtype-2 had more active immune reactions than subtype-1 overall (Figure 6B). Chemokines, cytokines, interferons, and interleukins were active in subtype-2. Meanwhile, similar trends were determined in the expression of HLA (Figure 6C). More infiltrating immunocytes, more active immune reactions, and higher HLA gene expression suggested that subtype-2 possessed immune enrichment.

### Identification of biological functions of distinct autophagy expression patterns

**FIGURE 7:**
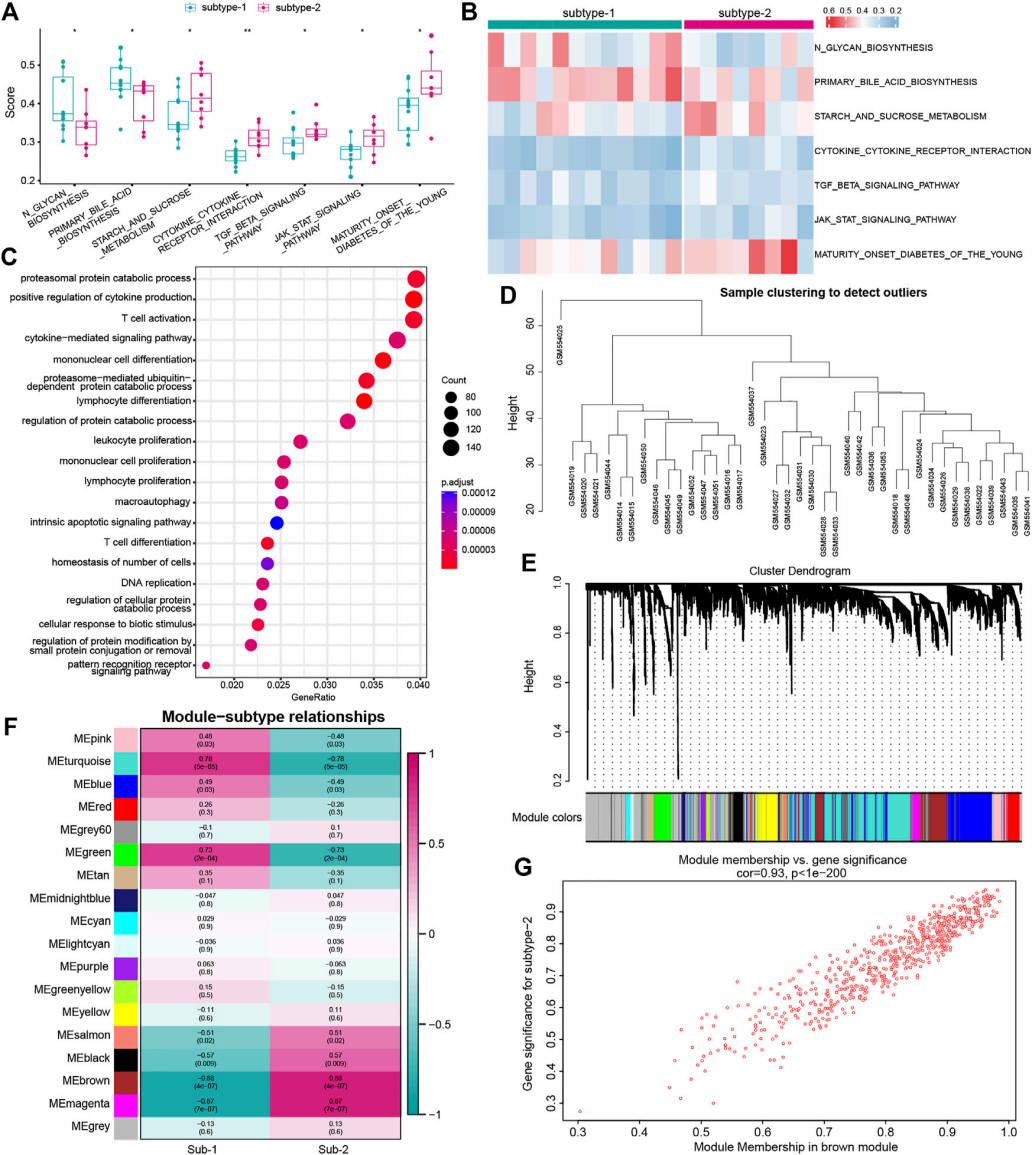
Underlying biological function characteristics of distinct autophagy expression subtypes. **(A)** Box plot showed the differences of the KEGG pathway enrichment score between subtype-1 and subtype-2 (*p* < 0.05). **(B)** Expression status of seven KEGG pathways in subtype-1 and subtype-2. **(C)** Top 20 of GO-BP enrichment analysis of autophagy phenotype-related genes. **(D)** Sample clustering was performed according to the expression data on all samples. The top 25% of variation genes were used for the analysis by WGCNA. **(E)** Gene dendrogram obtained by average linkage hierarchical clustering. The color row underneath the dendrogram shows the module assignment determined by the Dynamic Tree Cut, in which 18 modules were identified. **(F)** Heatmap showed the correlation between module eigengenes and the two autophagy expression patterns. **(G)** Scatterplot of gene significance (GS) for subtype-2 *versus* the module membership (MM) in the brown module. GS and MM exhibit a very significant correlation, implying that hub genes of the brown module also tend to be highly correlated with subtype-2.

To investigate the difference in biological functions between the two subtypes, GSVA analysis was applied to evaluate the enrichment score of the KEGG pathway (Figures 7A,B). We found that several important pathways were enriched in subtype-2, such as the cytokine–cytokine receptor interaction, TGF- β signaling pathway, and JAK-STAT signaling pathway. To further characterize the regulatory roles of distinct autophagy expression patterns, we first determined 5481 autophagy phenotype-related genes, which were differentially expressed between the two subtypes. GO-BP enrichment analysis of autophagy phenotype-related genes was then performed, and the result revealed that 30.6% (137/447) GO-BPs were remarkably related to immunity, such as lymphocyte differentiation, mononuclear cell differentiation, and T-cell activation or differentiation (Figure 7C; Supplementary Table S4). Next, we constructed a comprehensive gene landscape related to each autophagy expression pattern and identified gene–gene modules correlated with distinct autophagy regulations using the WGCNA analysis (Figures 7D,E, Supplementary Figure S5). A total of 18 gene modules were acquired, and the related genes were matched with different autophagy expression patterns (Figure 7F), such as autophagy subtype-2 being closely associated with genes in the brown module (Figure 7G). These results might have crucial implications for the gene expression regulation network mediated by autophagy.

### Validation of differentially expressed autophagy genes

Finally, differentially expressed analysis was performed on another two profiles (GSE58294 and GSE16561) for the validation of 20 dysregulated autophagy genes. As a result, 125 and 94 dysregulated autophagy genes were obtained in GSE58294 and GSE16561 datasets, respectively. A total of five autophagy genes, including *FOS*, *MAP1LC3B*, *ERO1L*, *ARNT*, and *PPP1R15A*, were found to be the intersection genes of the three lists of dysregulated autophagy genes (Supplementary Figure S6).

## Discussion

Autophagy is a core molecular pathway in eukaryotic cells for the preservation of cellular and organismal homeostasis. Under stressful conditions such as hypoxia, starvation, infection, and nutrient deficiencies, autophagy can be activated to provide nutrients and energy for the cells (Mo et al., 2020). Autophagy is a fundamental pathway for immunity, working in four ways, including the direct elimination of microorganisms, the regulation of inflammation, the regulation of innate immunity, and the regulation of adaptive immunity (Levine et al., 2011). It is reported that autophagy plays an important role in the clearance of protein aggregates caused by ischemia-induced neuronal endoplasmic reticulum stress (Liu et al., 2010). On the other hand, the immune system, as a critical part of the body, has been found to participate in the pathogenesis of IS. Considering that autophagy is closely related to immunity, it is believed that autophagy must have a momentous impact on the immune characteristics of IS. Therefore, we aim to excavate the changes in immunity in IS from a new perspective of autophagy to explore how it affects the immune characteristics of IS.

To address this point, we systematically identified the autophagy expression patterns related to the immune characteristics of IS. To further clarify the impact of autophagy on immune characteristics, including infiltrating immunocytes, immune reaction activity, and HLA gene expression, a series of bioinformatic algorithms were conducted, and the following important discoveries were obtained. First, 20 dysregulated autophagy genes were identified in IS, and the expression correlation and interaction among these genes implied a closely linked regulatory network involved in autophagy in IS. To test the ability of autophagy genes to distinguish IS samples, we performed the univariate logistic regression analysis, LASSO regression analysis, and multivariate regression analysis and constructed a classifier consisting of seven IS-related autophagy genes. The result of the ROC curve confirmed that the autophagy-related classifier has excellent discrimination ability. In clinical work, we usually used the TOAST classification to determine the etiologic subtypes of ischemic stroke that are based on the patient’s neurological signs, results of brain imaging, and findings of ancillary diagnostic tests. According to the TOAST classification, ischemic stroke is divided into five groups: large artery atherosclerosis (LAA), small vessel disease (SVD), cardio embolic disease (CE), other determined etiology, and undetermined etiology. However, information on clinical characteristics in etiologic stroke subtypes is scant. Through reviewing the publication (Tan et al., 2018), we found that the neurological deficit on admission differed significantly between etiologic subtypes. The most severe cases were CE patients, and the mildest cases were SVD patients. Another finding is that in-hospital complications such as respiratory infection, congestive heart failure, arrhythmia, and fervescence were most common in patients with CE and rare in patients with SAD. Of course, the autophagy genes might be different among the five groups. Therefore, it may be essential to investigate the relationship between the autophagy genes and IS based on the TOAST classification. Second, to figure out the immune characteristics of IS, the ssGSEA algorithm was used to evaluate the abundance of immune cells and the activity of immune reactions by establishing a matrix, and the expression of HLA genes was also taken into consideration. Their correlations with autophagy genes were then comprehensively analyzed. As a result, we found that the expression of NAMPT was dramatically positively correlated with mast cells, and ARNT was negatively correlated with activated CD4 T cells. NAMPT (nicotinamide phosphoribosyltransferase, also known as visfatin/pre-B-cell colony-enhancing factor) is a multifunctional protein and plays important roles in immunity, metabolism, inflammation, and stress responses. Lu et al. (2009) reported that the plasma NAMPT concentrations were increased in patients with IS. In a transient cerebral ischemia of a rat model, a significant increase in the thalamic mast cell number after 24 h from the ischemic insult has been observed (Hu et al., 2004). Mast cells serving as the first immune sentinel cells have been confirmed to exert critical roles in the pathogenesis of IS. These studies showed both NAMPT and mast cells presented a growing trend after cerebral ischemia, which supported our result. Meanwhile, we also found that PPP1R15A was positively correlated with chemokines, and CASP3 was negatively correlated with cytokines; NAMPT was positively correlated with HLAF, and ATG16L2 was negatively correlated with HLADOB. Some of these correlations have not been investigated in any other previous publications yet, which suggests that they might open a new insight to explore the role of autophagy in IS. Due to a close correlation that was found between autophagy and immunity in IS, we speculated different autophagy expression patterns might display diverse immune characteristics. Next, cluster analysis of IS samples was performed according to the autophagy expression profile, and we obtained two distinct expression patterns. The findings proved the opinion that the two subtypes emerged with differences in terms of immunocyte composition, immune reactions, and HLA gene expression. For example, higher fractions of γδ -T cells were observed in subtype-1, and it has been reported that γδ -T cells played a pro-inflammatory role in the pathophysiology of IS by releasing cytokines such as IL-17a, IL-21, IL-22, and IFN-γ (Wang et al., 2022). Additionally, significant changes in interferons and their receptors were identified between the two subtypes. Interferon‐β (IFN-β), a broadly expressed cytokine, drives innate immunity, acting in response to a pathogenic attack or injury *via* the activation of both pro- and anti-inflammatory cytokines (Wanve et al., 2019). A study reported that IFN-β could reduce the infarct size in ischemic brains and lessen neurological deficits in ischemic stroke animals (Kuo et al., 2016), which indicated a protective role of IFN‐β in IS. The two autophagy subtypes harboring diverse immune features hinted that autophagy might involve the immune process of IS, and they further exerted a critical role in the development of IS together. Furthermore, to uncover the biological features that cause the differences between the two autophagy expression patterns, the GSVA algorithm and GO-BP functional enrichment analysis were performed based on the two subtype expression profiles and autophagy phenotype-related genes, respectively. Interestingly, we found that the most significantly enriched signaling pathway is the cytokine–cytokine receptor interaction, and many of GO-BPs were also associated with immune response. In addition, gene modules related to autophagy-mediated expression patterns were identified using the WGCNA analysis. Finally, the expressions of the dysregulated autophagy genes were validated by two other datasets. The expression level differences of *FOS*, *MAP1LC3B*, *ERO1L*, *ARNT*, and *PPP1R15A* between the IS samples and healthy samples were proved. These findings indicated that autophagy indeed played a crucial role in the pathogenesis of IS. We further found that *FOS* and *MAP1LC3B* were also members of the autophagy-related classifier. Nevertheless, only 5 of the 20 dysregulated autophagy genes were differentially expressed in the other two datasets. We guessed that sampling at different time points in the course of ischemic stroke might have contributed to the difference in results. Additionally, a limitation to our study is that we only focused on the performance of the autophagy classifier in the IS samples. We should check the accuracy of the classifier in other datasets, which is unrelated to IS, especially those related to autophagy. Another limitation is that the GSE22255 dataset only contains 20 IS samples, which cannot represent the whole population. The course of IS is also a dynamic development process during which the expression status of autophagy genes may change. Therefore, the autophagy classifier may only be applicable to some IS samples with the same disease characteristics. Whether the autophagy classifier can be applied to all the IS samples needs to be further validated by expanding the sample size.

Our work systematically investigated the relationship between autophagy and immune characteristics in IS for the first time. In the study, some findings that were reported in other diseases or fire-new and required attention was obtained, and the findings might contribute to enlightening the development of immunotherapy from the view of autophagy in IS. Furthermore, the two distinct autophagy expression patterns we identified were different from any other classification standards of IS, which could help us improve the understanding of autophagy in IS and how it affects immunity. Taken together, there might be a deep connection between autophagy and immune characteristics, and it is believed that these findings will promote researchers to further explore the roles of autophagy in IS and gradually reveal the harboring molecule mechanism of IS.

## Conclusion

In conclusion, our work uncovered the potential mechanism of the impact of autophagy on the immunity of IS. The comprehensively systematic investigation of autophagy expression patterns will provide a novel perspective for understanding the pathogenesis of IS and open a new direction for researchers to explore the molecular mechanism in other diseases.

## Data Availability

The datasets presented in this study can be found in online repositories. The names of the repository/repositories and accession number(s) can be found in the article/Supplementary Material.

## Supplementary material

SUPPLEMENTARY FIGURE S1 Validation of autophagy-related classifier discrimination ability using the GSE58294 dataset. **(A)** Risk distribution between IS and healthy subjects, where IS has a much higher risk score than healthy samples. **(B)** ROC curve evaluated the discrimination ability of the classifier model.

SUPPLEMENTARY FIGURE S2 Difference in the abundance of each immunocyte cell between healthy and IS samples.

SUPPLEMENTARY FIGURE S3 Difference in the immune reaction activity between healthy and IS samples.

SUPPLEMENTARY FIGURE S4 Difference in the expression of each HLA gene between healthy and IS samples.

SUPPLEMENTARY FIGURE S5 Analysis of the scale-free ft index and analysis of the mean connectivity for various soft-thresholding powers.

SUPPLEMENTARY FIGURE S6 Validation of 20 dysregulated autophagy genes.
