# Supplementary figures and images for: Identification of immune characteristic landscapes related to autophagy in ischemic stroke
Source: Front Cell Dev Biol. 2022 Nov 29;10:1026578. doi: 10.3389/fcell.2022.1026578 (PMC9745074; doi:10.3389/fcell.2022.1026578)

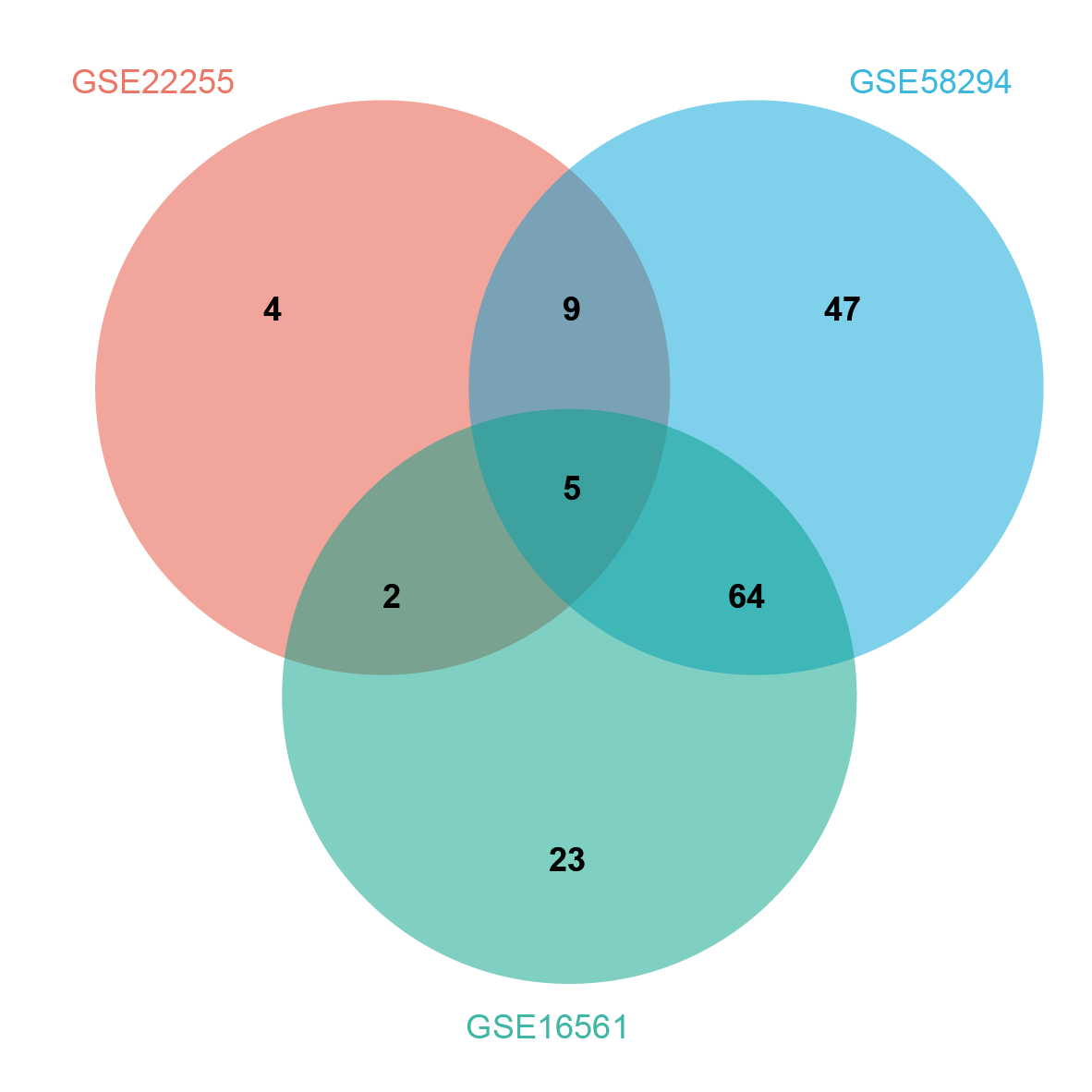

Supplement: Supplementary file 3 [file Image6.TIF]

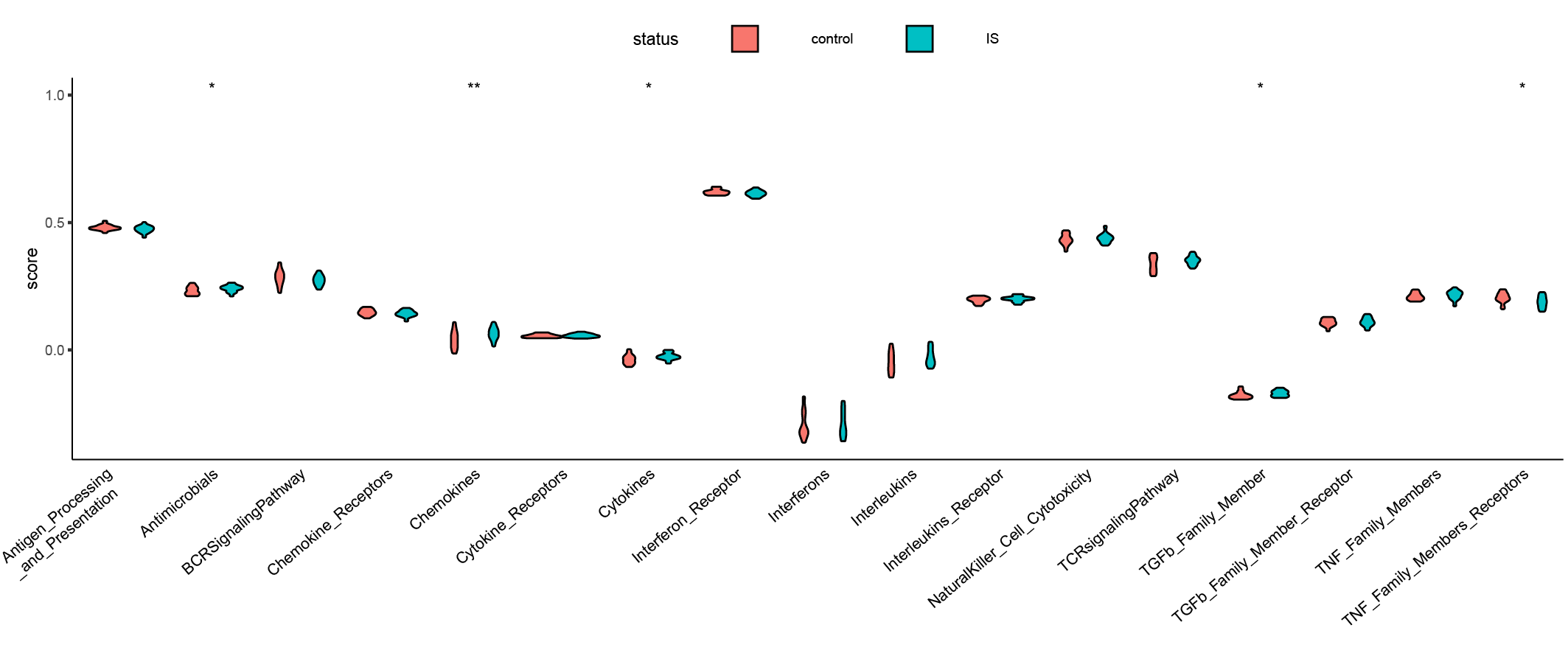

Supplement: Supplementary file 4 [file Image3.TIF]

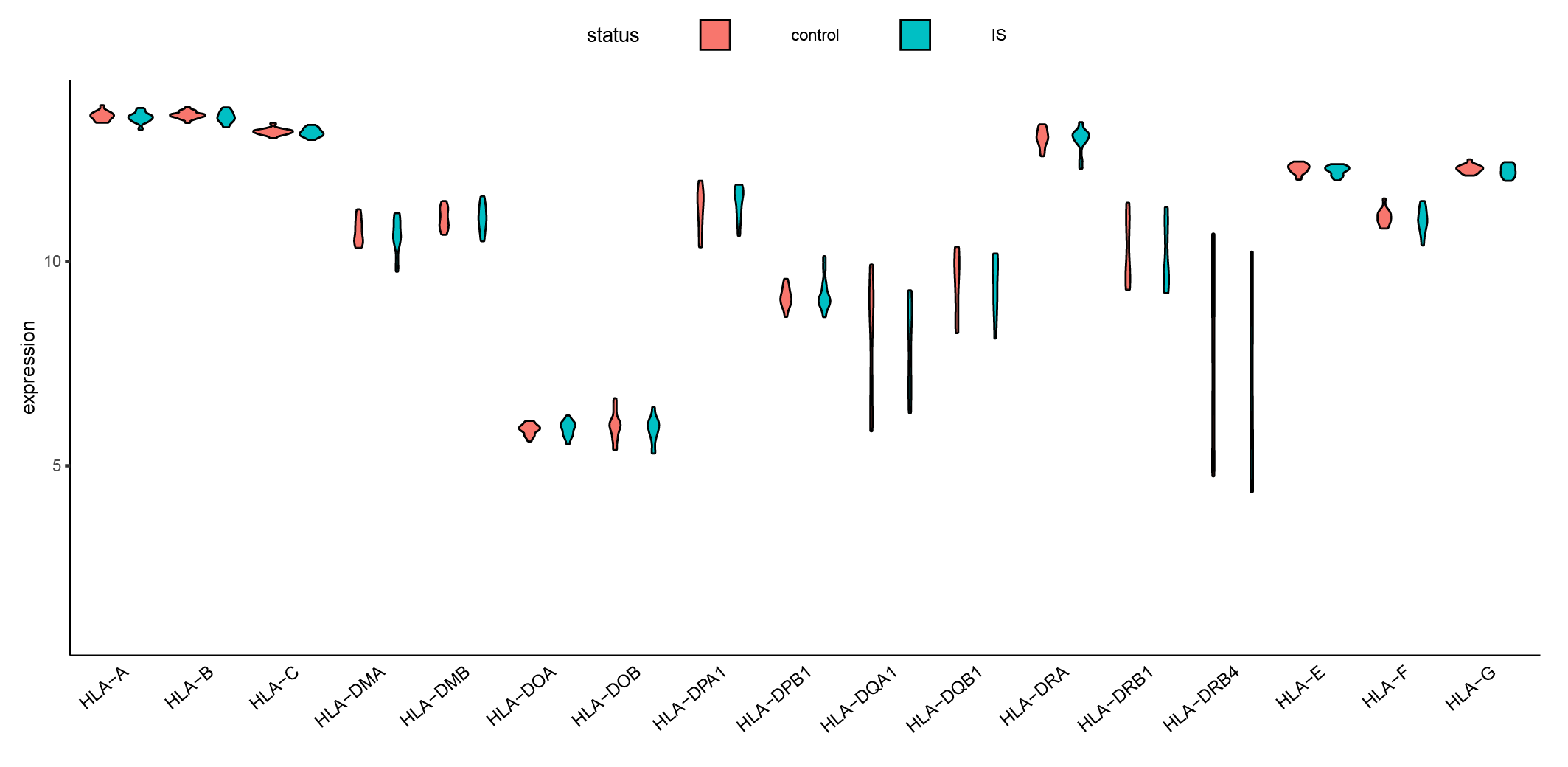

Supplement: Supplementary file 5 [file Image4.TIF]

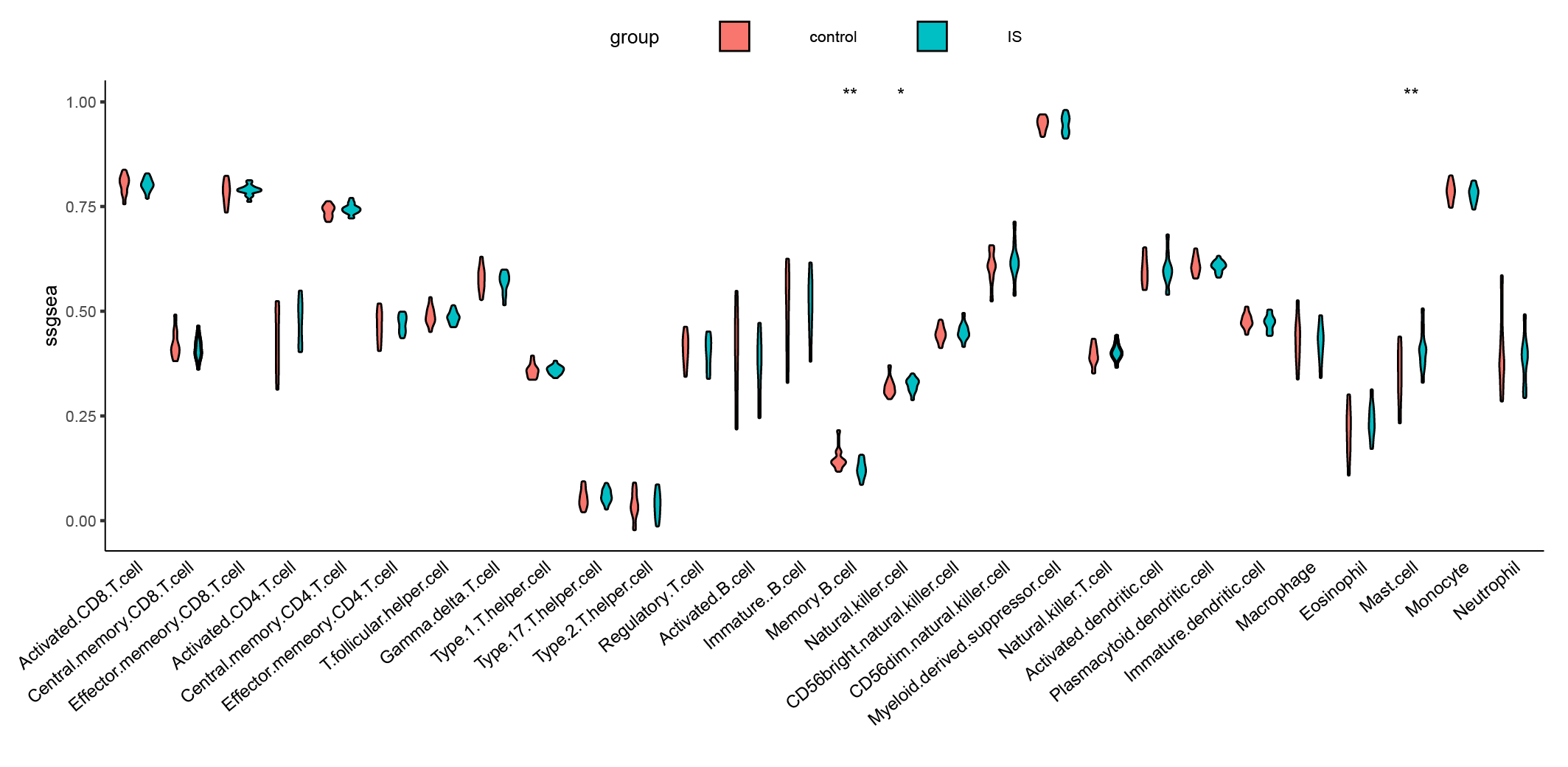

Supplement: Supplementary file 6 [file Image2.TIF]

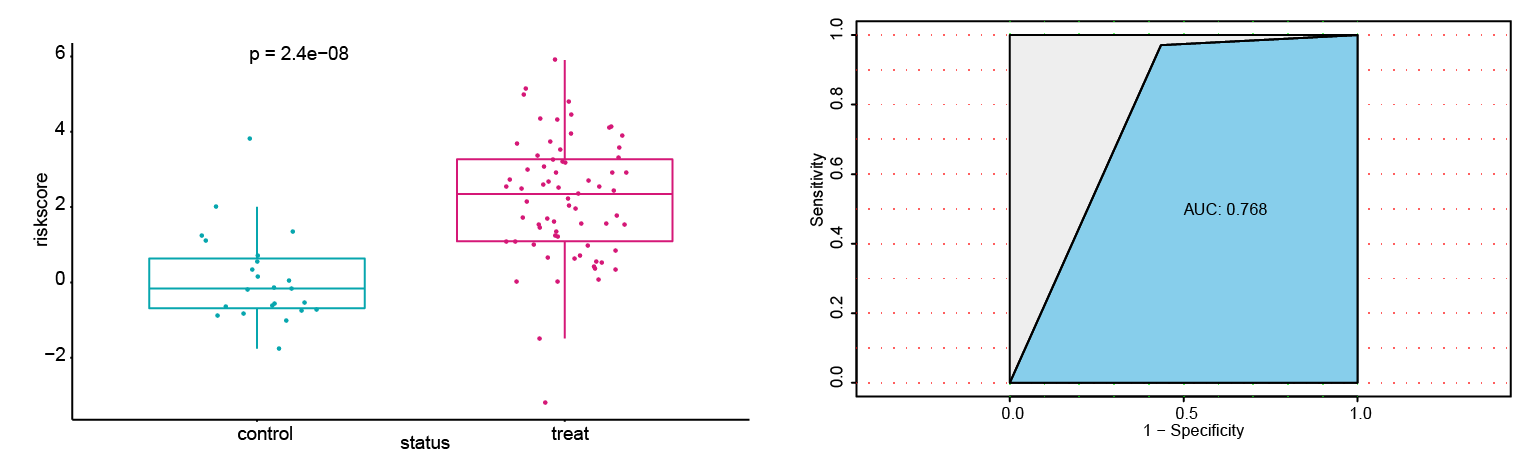

Supplement: Supplementary file 7 [file Image1.TIF]

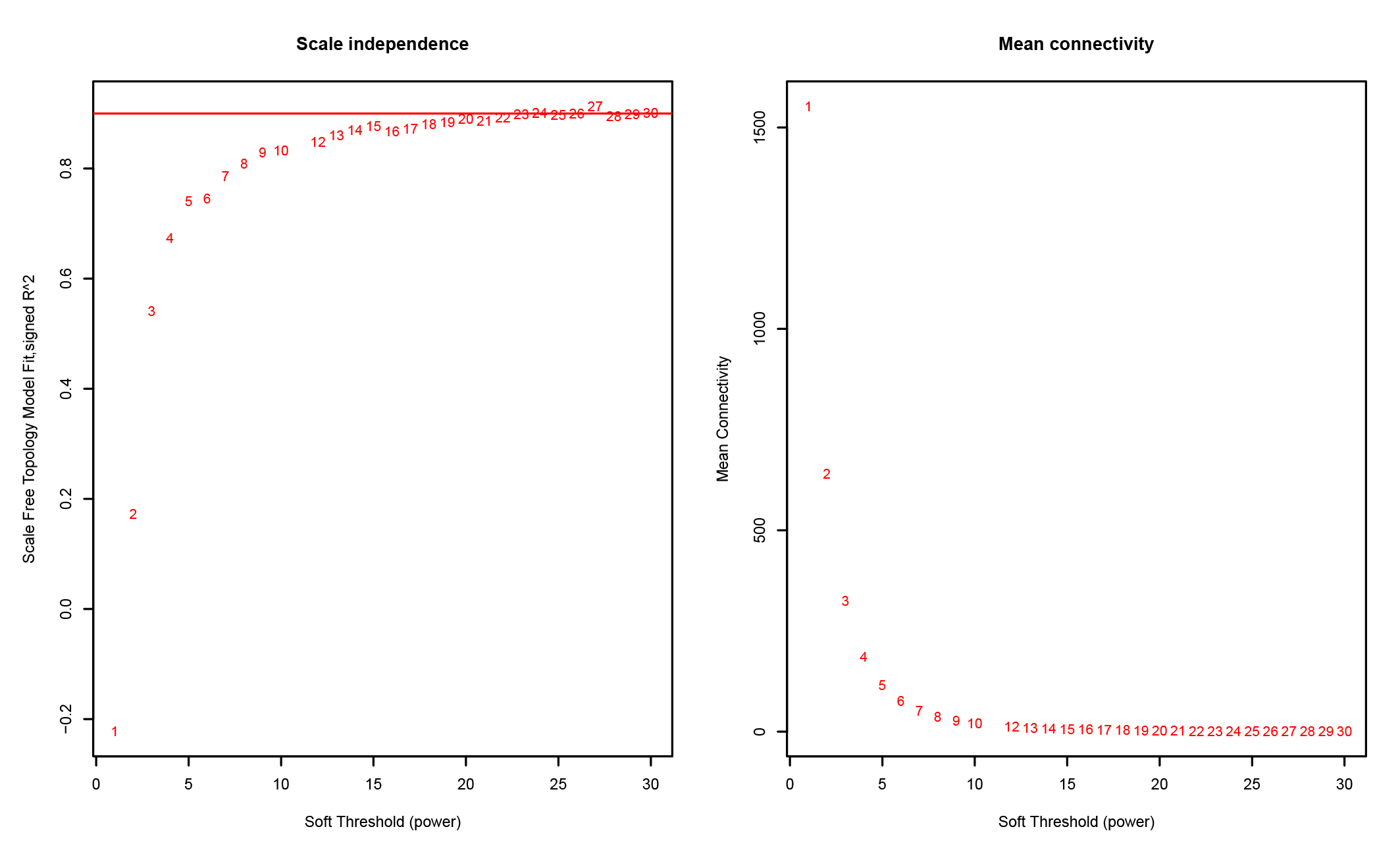

Supplement: Supplementary file 10 [file Image5.TIF]
